# (Propane-1,3-diammonium) bis­(4-hydroxy­pyridine-2,6-dicarboxyl­ato-κ^3^ *O* ^2^,*N*,*O* ^6^)zinc(II) 3.5-hydrate

**DOI:** 10.1107/S1600536809041634

**Published:** 2009-10-17

**Authors:** Mohammad Ghadermazi, Faranak Manteghi, Hossein Aghabozorg

**Affiliations:** a Department of Chemistry, Faculty of Science, University of Kurdistan, Sanandaj, Iran; b Department of Chemistry, Iran University of Science and Technology, Tehran, Iran; c Faculty of Chemistry, Islamic Azad University, North Tehran Branch, Tehran, Iran

## Abstract

The asymmetric unit of the title compound, (C_3_H_12_N_2_)[Zn(C_7_H_3_NO_5_)_2_]·3.5H_2_O, contains two formula units. The compound consists of an anionic complex, a doubly protonated propane-1,3-diamine as a counter-ion and 3.5 uncoord­inated water mol­ecules. The coordination polyhedron around the Zn^II ^atom is distorted octa­hedral, defined by four O atoms and two N atoms from two Hchel (H_3_chel = 4-hydroxy­pyridine-2,6-dicarboxylic acid) ligands. In the crystal structure, O—H⋯O, N—H⋯O and C—H⋯O hydrogen bonds along with π–π stacking inter­actions [centroid–centroid distance = 3.473 (2) Å] are observed to reinforce the crystal cohesion.

## Related literature

For related structures, see: Aghabozorg *et al.* (2007 ▶, 2008*a* ▶,*b* ▶); Hall *et al.* (2000 ▶); Ramos Silva *et al.* (2008 ▶); Soleimannejad *et al.* (2009 ▶); Zhou *et al.* (2003 ▶, 2007 ▶); Zou *et al.* (2009 ▶).
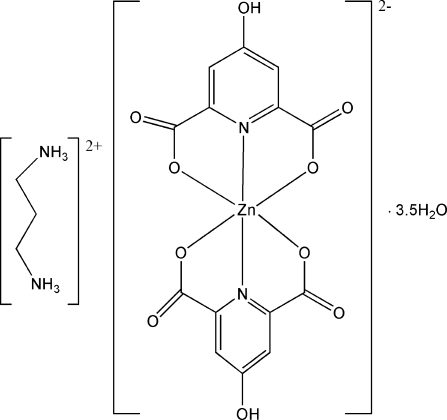


## Experimental

### 

#### Crystal data

(C_3_H_12_N_2_)[Zn(C_7_H_3_NO_5_)_2_]·3.5H_2_O
*M* *_r_* = 566.78
Monoclinic, 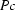

*a* = 11.853 (3) Å
*b* = 12.633 (2) Å
*c* = 15.333 (3) Å
β = 98.93 (3)°
*V* = 2268.1 (8) Å^3^
*Z* = 4
Mo *K*α radiation
μ = 1.16 mm^−1^
*T* = 120 K
0.25 × 0.20 × 0.10 mm

#### Data collection

Bruker SMART 1000 CCD diffractometer
Absorption correction: multi-scan (*SADABS*; Sheldrick, 1996 ▶) *T*_min_ = 0.757, *T*_max_ = 0.891
24459 measured reflections
11831 independent reflections
9079 reflections with *I* > 2σ(*I*)
*R*_int_ = 0.037

#### Refinement

*R*[*F* ^2^ > 2σ(*F* ^2^)] = 0.039
*wR*(*F* ^2^) = 0.080
*S* = 1.00
11831 reflections
659 parameters
14 restraints
H atoms treated by a mixture of independent and constrained refinement
Δρ_max_ = 0.61 e Å^−3^
Δρ_min_ = −0.38 e Å^−3^
Absolute structure: Flack (1983 ▶), 5792 Friedel pairs
Flack parameter: 0.492 (8)

### 

Data collection: *SMART* (Bruker, 2007 ▶); cell refinement: *SAINT-Plus* (Bruker, 2007 ▶); data reduction: *SAINT-Plus*; program(s) used to solve structure: *SHELXTL* (Sheldrick, 2008 ▶); program(s) used to refine structure: *SHELXTL*; molecular graphics: *SHELXTL*; software used to prepare material for publication: *SHELXTL*.

## Supplementary Material

Crystal structure: contains datablocks I, global. DOI: 10.1107/S1600536809041634/hy2228sup1.cif

Structure factors: contains datablocks I. DOI: 10.1107/S1600536809041634/hy2228Isup2.hkl

Additional supplementary materials:  crystallographic information; 3D view; checkCIF report

## Figures and Tables

**Table 1:** Selected bond lengths (Å)

Zn1—N1	2.012 (3)
Zn1—N2	1.999 (3)
Zn1—O1	2.081 (3)
Zn1—O3	2.424 (3)
Zn1—O6	2.155 (3)
Zn1—O8	2.201 (3)
Zn2—N3	2.001 (3)
Zn2—N4	2.007 (3)
Zn2—O11	2.331 (2)
Zn2—O13	2.135 (3)
Zn2—O16	2.234 (3)
Zn2—O18	2.149 (3)

**Table 2:** Hydrogen-bond geometry (Å, °)

*D*—H⋯*A*	*D*—H	H⋯*A*	*D*⋯*A*	*D*—H⋯*A*
O5—H5*O*⋯O1*W*^i^	0.82 (4)	1.79 (4)	2.577 (5)	162 (3)
O10—H10*O*⋯O12^ii^	0.89	1.73	2.613 (4)	170
O15—H15*O*⋯O2*W*^iii^	0.88 (4)	1.73 (4)	2.600 (4)	170 (4)
O20—H20*O*⋯O4	0.85	1.80	2.600 (3)	156
N5—H5*NA*⋯O14^iv^	0.86	2.16	3.019 (4)	176
N5—H5*NB*⋯O4*W*	0.86	1.94	2.804 (4)	176
N5—H5*NC*⋯O4^v^	0.86	2.23	3.019 (4)	152
N6—H6*NA*⋯O14^vi^	0.86	2.03	2.870 (4)	164
N6—H6*NB*⋯O5*W*^i^	0.86	1.94	2.796 (3)	174
N6—H6*NC*⋯O9^vii^	0.86	1.93	2.771 (4)	164
N7—H7*NA*⋯O16^vi^	0.86	2.15	2.943 (4)	153
N7—H7*NB*⋯O19^iv^	0.86	1.99	2.714 (4)	141
N7—H7*NC*⋯O9^vii^	0.86	2.14	2.991 (4)	169
N8—H8*NA*⋯O6*W*^vi^	0.86	1.89	2.724 (4)	164
N8—H8*NB*⋯O2^vii^	0.86	2.00	2.828 (4)	160
N8—H8*NC*⋯O3*W*^viii^	0.86	1.94	2.791 (4)	169
O1*W*—H1*WA*⋯O18^ix^	0.82 (4)	1.90 (4)	2.701 (4)	164 (4)
O1*W*—H1*WB*⋯O1	0.86 (3)	1.84 (4)	2.690 (4)	171 (4)
O2*W*—H2*WA*⋯O8	0.83 (4)	1.94 (4)	2.738 (4)	163 (3)
O2*W*—H2*WB*⋯O13^x^	0.89 (3)	1.81 (3)	2.688 (4)	171 (3)
O3*W*—H3*WA*⋯O17^i^	0.82	2.05	2.873 (4)	179
O3*W*—H3*WB*⋯O3^i^	0.82	2.14	2.950 (3)	172
O4*W*—H4*WA*⋯O20	0.82	2.32	2.876 (4)	126
O4*W*—H4*WB*⋯O11^v^	0.82	2.06	2.862 (3)	167
O5*W*—H5*WA*⋯O7	0.82	1.94	2.717 (3)	158
O5*W*—H5*WB*⋯O17^i^	0.82	2.05	2.846 (3)	165
O6*W*—H6*WA*⋯O11^ii^	0.82	1.94	2.755 (4)	169
O6*W*—H6*WB*⋯O7	0.82	1.96	2.726 (3)	154
O7*W*—H7*WA*⋯O3	0.82	2.03	2.829 (3)	164
O7*W*—H7*WB*⋯O17	0.82	2.14	2.953 (3)	170
C12—H12*A*⋯O6^xi^	0.93	2.33	3.209 (5)	157
C32—H32*B*⋯O2^i^	0.97	2.37	3.213 (4)	145

Symmetry codes: (i) 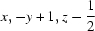; (ii) 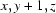; (iii) 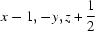; (iv) 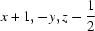; (v) 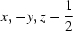; (vi) 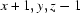; (vii) 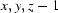; (viii) 
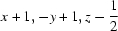
; (ix) 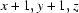; (x) 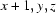; (xi) 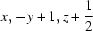.

**Fig. 1.**
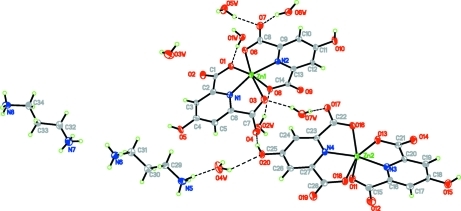
Molecular structure of the title compound. Displacement ellipsoids are drawn at the 50% probability level. Dashed lines denote hydrogen bonds.

**Fig. 2.**
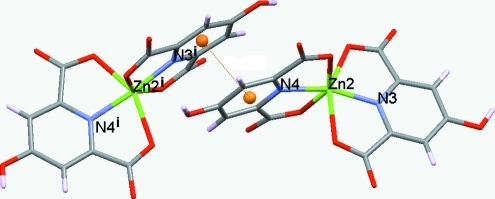
The π–π stacking interaction (dotted line), with centroid–centroid distance of 3.473 (2) Å. [Symmetry code: (i) x, -y, -1/2+z.]

**Fig. 3.**
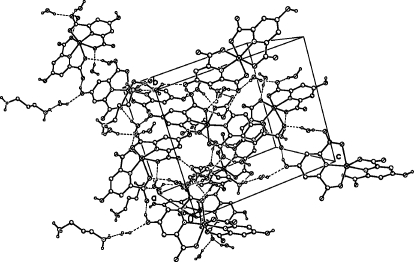
The crystal packing of the title compound.

## supplementary crystallographic information

### Comment

There are several reports on coordination of 4-hydroxypyridine-2,6-dicarboxylic acid (H_3_chel) to metals, such as [Fe(Hchel)Cl(H_2_O)_2_](18-crown-6).2H_2_O (Zhou *et al.*, 2007), [Cu(Hchel)(dmp].3H_2_O (Soleimannejad *et al.*, 2009), Na_5_[Gd(chel)_2_(Hchel)].16H_2_O (Hall *et al.*, 2000) and (tataH)_2_[Cu(Hchel)_2_.6H_2_O (tata = 2,4,6-triamino-1,3,5-triazine) (Ramos Silva *et al.*, 2008), (GH)_2_[Ni(Hchel)_2_].2H_2_O (G = guanidine) (Aghabozorg *et al.*, 2008b), [Zn(chel)(H_2_O)_3_].0.25CH_3_CN.H_2_O (Zhou *et al.*, 2003), [Zn_3_(chel)_2_.8H_2_O]*_n_* (Zou *et al.*, 2009), [Zn_12_(Hchel)_12_(H_2_O)_10_.12H_2_O]*_n_* (Aghabozorg *et al.*, 2007), and the most similar to the title compound, (pnH_2_)[Hg(Hchel)Cl(H_2_O)]_2_.4H_2_O (pn = propane-1,3-diamine) (Aghabozorg *et al.*, 2008a).

The title compound has two molecules in the asymmetric unit (Fig. 1). All Zn—O and Zn—N bond lengths and angles are in normal range (Table 1). In the asymmetric unit, the two complexes slightly differ in bond lengths and bond angles. Both complexes have a six-coordinated octahedral geometry around the Zn^II^ atom. The N1—Zn1—N2 and N3—Zn2—N4 angles, 157.03 (13) and 160.29 (12)°, respectively, are the nearest angles to linearity, so it can be concluded that the N atoms of the two Hchel ligands locate at the axial positions, and the other atoms at the equatorial positions of the distorted octahedral geometry.

The crystal structure contains many O—H···O, N—H···O and C—H···O hydrogen bonds, shown in Table 2. The π–π stacking with centroid–centroid distance of 3.473 (2) Å is illustrated in Fig. 2. The crystal packing is shown in Fig. 3.

### Experimental

From a solution of propane-1,3-diamine (0.029 g, 0.4 mmol) and 4-hydroxypyridine-2,6-dicarboxylic acid (0.073 g, 0.4 mmol) in THF (30 ml), a white precipitate was obtained. By mixing the precipitate with Zn(NO_3_)_2_.6H_2_O (0.052 g, 0.2 mmol) in water (25 ml) and heating for 2 h at 333 K, colourless crystals of the title compound were obtained after allowing the mixture to stand for four weeks at room temperature.

### Refinement

H atoms of OH and NH_3_ groups and water molecules were found in difference Fourier maps. The H atoms bound to O5, O15, O1W and O2W were refined with *U*_iso_(H) = 1.2*U*_eq_(O), and the others were refined as riding atoms, with *U*_iso_(H) = 1.2*U*_eq_(O,N). H atoms on C atoms were positioned geometrically and refined as riding, with C—H = 0.93 (aromatic) and 0.97 (CH_2_) Å and with *U*_iso_(H) = 1.2*U*_eq_(C).

### Crystal data

**Table:**

(C_3_H_12_N_2_)[Zn(C_7_H_3_NO_5_)_2_]·3.5H_2_O	*F*(000) = 1172
*M**_r_* = 566.78	*D*_x_ = 1.660 Mg m^−^^3^
Monoclinic, *P**c*	Mo *K*α radiation, λ = 0.71073 Å
Hall symbol: P -2yc	Cell parameters from 587 reflections
*a* = 11.853 (3) Å	θ = 3–29°
*b* = 12.633 (2) Å	µ = 1.16 mm^−^^1^
*c* = 15.333 (3) Å	*T* = 120 K
β = 98.93 (3)°	Plate, colorless
*V* = 2268.1 (8) Å^3^	0.25 × 0.20 × 0.10 mm
*Z* = 4	

### Data collection

**Table:**

Bruker SMART 1000 CCD diffractometer	11831 independent reflections
Radiation source: fine-focus sealed tube	9079 reflections with *I* > 2σ(*I*)
graphite	*R*_int_ = 0.037
φ and ω scans	θ_max_ = 29.0°, θ_min_ = 1.6°
Absorption correction: multi-scan (*SADABS*; Sheldrick, 1996)	*h* = −16→16
*T*_min_ = 0.757, *T*_max_ = 0.891	*k* = −17→17
24459 measured reflections	*l* = −20→20

### Refinement

**Table:**

Refinement on *F*^2^	Secondary atom site location: difference Fourier map
Least-squares matrix: full	Hydrogen site location: mixed
*R*[*F*^2^ > 2σ(*F*^2^)] = 0.039	H atoms treated by a mixture of independent and constrained refinement
*wR*(*F*^2^) = 0.080	*w* = 1/[σ^2^(*F*_o_^2^) + (0.026*P*)^2^ + 0.24*P*] where *P* = (*F*_o_^2^ + 2*F*_c_^2^)/3
*S* = 1.00	(Δ/σ)_max_ = 0.001
11831 reflections	Δρ_max_ = 0.61 e Å^−^^3^
659 parameters	Δρ_min_ = −0.38 e Å^−^^3^
14 restraints	Absolute structure: Flack (1983), 5792 Friedel pairs
Primary atom site location: structure-invariant direct methods	Flack parameter: 0.492 (8)

### Fractional atomic coordinates and isotropic or equivalent isotropic displacement parameters (Å^2^)

**Table:**

	*x*	*y*	*z*	*U*_iso_*/*U*_eq_	
Zn1	0.50235 (3)	0.46750 (3)	0.88270 (2)	0.01608 (10)	
O1	0.6535 (2)	0.53464 (18)	0.85562 (18)	0.0177 (6)	
O2	0.7867 (2)	0.5165 (2)	0.76790 (18)	0.0218 (6)	
O3	0.3643 (2)	0.32454 (19)	0.86439 (16)	0.0219 (6)	
O4	0.3500 (2)	0.16616 (18)	0.79754 (15)	0.0236 (5)	
O5	0.5712 (3)	0.2441 (2)	0.54295 (18)	0.0228 (6)	
H5O	0.613 (3)	0.272 (3)	0.512 (2)	0.027*	
N1	0.5313 (2)	0.3803 (2)	0.77863 (19)	0.0145 (7)	
C1	0.6915 (3)	0.4956 (3)	0.7887 (2)	0.0159 (8)	
C2	0.6169 (3)	0.4132 (3)	0.7370 (3)	0.0155 (8)	
C3	0.6351 (3)	0.3701 (3)	0.6581 (2)	0.0154 (8)	
H3A	0.6946	0.3943	0.6304	0.018*	
C4	0.5629 (3)	0.2894 (3)	0.6200 (2)	0.0170 (8)	
C5	0.4795 (3)	0.2504 (3)	0.6674 (2)	0.0172 (8)	
H5A	0.4339	0.1933	0.6461	0.021*	
C6	0.4662 (3)	0.2980 (3)	0.7460 (2)	0.0151 (8)	
C7	0.3843 (3)	0.2600 (3)	0.8063 (2)	0.0191 (8)	
O6	0.3962 (2)	0.59727 (19)	0.82811 (16)	0.0189 (6)	
O7	0.2541 (2)	0.7022 (2)	0.85680 (17)	0.0221 (6)	
O8	0.5893 (2)	0.3753 (2)	0.99522 (17)	0.0181 (6)	
O9	0.5808 (2)	0.3416 (2)	1.13726 (17)	0.0193 (6)	
O10	0.2270 (2)	0.5656 (2)	1.17553 (17)	0.0249 (6)	
H10O	0.1725	0.6130	1.1589	0.030*	
N2	0.4233 (3)	0.5118 (2)	0.9829 (2)	0.0144 (7)	
C8	0.3277 (3)	0.6312 (3)	0.8767 (2)	0.0175 (8)	
C9	0.3388 (3)	0.5825 (3)	0.9677 (2)	0.0146 (8)	
C10	0.2703 (3)	0.6058 (3)	1.0312 (2)	0.0158 (8)	
H10A	0.2137	0.6574	1.0215	0.019*	
C11	0.2901 (3)	0.5481 (3)	1.1109 (3)	0.0161 (8)	
C12	0.3777 (3)	0.4745 (3)	1.1256 (3)	0.0124 (8)	
H12A	0.3916	0.4364	1.1781	0.015*	
C13	0.4437 (3)	0.4593 (3)	1.0600 (3)	0.0141 (8)	
C14	0.5452 (3)	0.3849 (3)	1.0661 (3)	0.0141 (8)	
Zn2	−0.12188 (3)	−0.02728 (3)	1.01277 (2)	0.01528 (10)	
O11	0.0296 (2)	−0.14519 (19)	1.04823 (16)	0.0196 (6)	
O12	0.0786 (2)	−0.2812 (2)	1.14128 (17)	0.0319 (6)	
O13	−0.2797 (2)	0.0411 (2)	1.03501 (18)	0.0184 (6)	
O14	−0.4204 (2)	0.0173 (2)	1.11457 (18)	0.0206 (6)	
O15	−0.2106 (2)	−0.2439 (2)	1.35147 (18)	0.0237 (6)	
H15O	−0.269 (3)	−0.216 (3)	1.372 (2)	0.028*	
N3	−0.1529 (2)	−0.1050 (2)	1.1203 (2)	0.0146 (6)	
C15	0.0181 (3)	−0.2057 (3)	1.1134 (2)	0.0210 (8)	
C16	−0.0838 (3)	−0.1806 (3)	1.1597 (2)	0.0154 (8)	
C17	−0.1023 (3)	−0.2290 (3)	1.2367 (2)	0.0182 (8)	
H17A	−0.0533	−0.2816	1.2626	0.022*	
C18	−0.1958 (3)	−0.1973 (3)	1.2748 (2)	0.0168 (8)	
C19	−0.2690 (3)	−0.1203 (3)	1.2332 (2)	0.0167 (8)	
H19A	−0.3322	−0.0982	1.2575	0.020*	
C20	−0.2460 (3)	−0.0772 (3)	1.1549 (2)	0.0132 (8)	
C21	−0.3222 (3)	0.0008 (3)	1.0986 (3)	0.0155 (8)	
O16	−0.0287 (2)	0.1199 (2)	1.06223 (17)	0.0182 (6)	
O17	0.1104 (2)	0.2269 (2)	1.03114 (16)	0.0181 (6)	
O18	−0.1961 (2)	−0.1272 (2)	0.90562 (17)	0.0183 (6)	
O19	−0.1854 (2)	−0.1664 (2)	0.76526 (18)	0.0237 (6)	
O20	0.1724 (2)	0.0551 (2)	0.73170 (17)	0.0210 (6)	
H20O	0.2249	0.1016	0.7400	0.025*	
N4	−0.0427 (3)	0.0196 (2)	0.9128 (2)	0.0131 (7)	
C22	0.0418 (3)	0.1510 (3)	1.0141 (2)	0.0165 (8)	
C23	0.0400 (3)	0.0918 (3)	0.9267 (2)	0.0130 (8)	
C24	0.1146 (3)	0.1069 (3)	0.8686 (2)	0.0144 (8)	
H24A	0.1720	0.1576	0.8794	0.017*	
C25	0.1029 (3)	0.0445 (3)	0.7922 (3)	0.0153 (8)	
C26	0.0155 (3)	−0.0297 (3)	0.7771 (3)	0.0152 (8)	
H26A	0.0054	−0.0712	0.7264	0.018*	
C27	−0.0557 (3)	−0.0399 (3)	0.8396 (3)	0.0125 (8)	
C28	−0.1520 (3)	−0.1184 (3)	0.8355 (3)	0.0158 (8)	
N5	0.4016 (3)	−0.0146 (2)	0.4508 (2)	0.0209 (7)	
H5NA	0.4519	−0.0189	0.4976	0.025*	
H5NB	0.3527	0.0015	0.4847	0.025*	
H5NC	0.3881	−0.0730	0.4223	0.025*	
N6	0.4727 (2)	0.1754 (2)	0.20851 (17)	0.0222 (6)	
H6NA	0.4942	0.1204	0.1824	0.027*	
H6NB	0.4007	0.1783	0.2108	0.027*	
H6NC	0.4959	0.2343	0.1894	0.027*	
C29	0.4101 (3)	0.0808 (3)	0.3943 (2)	0.0207 (7)	
H29A	0.4104	0.1443	0.4299	0.025*	
H29B	0.3441	0.0837	0.3482	0.025*	
C30	0.5171 (3)	0.0768 (3)	0.3532 (2)	0.0222 (8)	
H30A	0.5134	0.0154	0.3150	0.027*	
H30B	0.5818	0.0673	0.3997	0.027*	
C31	0.5382 (3)	0.1748 (3)	0.2996 (2)	0.0213 (8)	
H31A	0.5179	0.2373	0.3306	0.026*	
H31B	0.6190	0.1791	0.2960	0.026*	
N7	0.8201 (3)	0.2833 (2)	0.1175 (2)	0.0263 (7)	
H7NA	0.8528	0.2421	0.0843	0.032*	
H7NB	0.8340	0.2738	0.1736	0.032*	
H7NC	0.7534	0.3085	0.1205	0.032*	
N8	0.9664 (3)	0.5324 (2)	−0.0880 (2)	0.0232 (7)	
H8NA	0.9821	0.5988	−0.0839	0.028*	
H8NB	0.9029	0.5212	−0.1223	0.028*	
H8NC	1.0192	0.4900	−0.0990	0.028*	
C32	0.8925 (3)	0.3776 (3)	0.1081 (2)	0.0256 (8)	
H32A	0.9713	0.3615	0.1320	0.031*	
H32B	0.8680	0.4359	0.1417	0.031*	
C33	0.8851 (3)	0.4100 (3)	0.0125 (2)	0.0211 (7)	
H33A	0.9140	0.3533	−0.0205	0.025*	
H33B	0.8058	0.4222	−0.0125	0.025*	
C34	0.9533 (3)	0.5094 (3)	0.0042 (2)	0.0227 (7)	
H34A	0.9156	0.5687	0.0277	0.027*	
H34B	1.0283	0.5018	0.0393	0.027*	
O1W	0.7384 (3)	0.6872 (2)	0.96956 (19)	0.0235 (6)	
H1WA	0.758 (3)	0.737 (3)	0.941 (3)	0.028*	
H1WB	0.714 (3)	0.642 (3)	0.929 (2)	0.028*	
O2W	0.6128 (2)	0.1819 (2)	0.91942 (18)	0.0229 (6)	
H2WA	0.602 (3)	0.233 (3)	0.951 (2)	0.027*	
H2WB	0.643 (3)	0.137 (3)	0.961 (2)	0.027*	
O3W	0.1322 (2)	0.62023 (18)	0.39603 (17)	0.0344 (6)	
H3WA	0.1263	0.6642	0.4344	0.041*	
H3WB	0.1958	0.6413	0.3894	0.041*	
O4W	0.2384 (2)	0.0285 (2)	0.56029 (16)	0.0341 (6)	
H4WA	0.2548	0.0046	0.6104	0.041*	
H4WB	0.1736	0.0526	0.5567	0.041*	
O5W	0.23614 (19)	0.81030 (18)	0.70209 (15)	0.0255 (5)	
H5WA	0.2315	0.7655	0.7401	0.031*	
H5WB	0.1893	0.8010	0.6575	0.031*	
O6W	0.0423 (2)	0.73460 (18)	0.90019 (17)	0.0248 (6)	
H6WA	0.0333	0.7759	0.9398	0.030*	
H6WB	0.0965	0.7339	0.8728	0.030*	
O7W	0.3576 (2)	0.21434 (19)	1.02412 (16)	0.0246 (6)	
H7WA	0.3553	0.2566	0.9833	0.030*	
H7WB	0.2913	0.2219	1.0322	0.030*	

### Atomic displacement parameters (Å^2^)

**Table:**

	*U*^11^	*U*^22^	*U*^33^	*U*^12^	*U*^13^	*U*^23^
Zn1	0.0172 (2)	0.0175 (2)	0.0147 (2)	−0.00004 (18)	0.00636 (18)	−0.00176 (18)
O1	0.0188 (15)	0.0194 (14)	0.0159 (15)	−0.0038 (11)	0.0052 (12)	−0.0028 (10)
O2	0.0157 (14)	0.0304 (15)	0.0201 (14)	−0.0037 (12)	0.0051 (11)	0.0000 (11)
O3	0.0209 (13)	0.0257 (14)	0.0201 (14)	−0.0004 (11)	0.0062 (11)	−0.0026 (11)
O4	0.0232 (13)	0.0202 (12)	0.0284 (13)	−0.0076 (10)	0.0066 (11)	−0.0002 (10)
O5	0.0300 (15)	0.0262 (14)	0.0145 (14)	−0.0010 (12)	0.0109 (11)	−0.0053 (11)
N1	0.0120 (15)	0.0170 (15)	0.0139 (16)	−0.0012 (12)	0.0002 (12)	0.0007 (12)
C1	0.021 (2)	0.0150 (17)	0.0120 (18)	0.0005 (15)	0.0040 (15)	0.0070 (15)
C2	0.0155 (19)	0.0157 (17)	0.0150 (19)	0.0014 (15)	0.0012 (15)	0.0042 (15)
C3	0.0195 (18)	0.0145 (17)	0.0131 (18)	0.0015 (14)	0.0056 (15)	−0.0007 (14)
C4	0.0175 (18)	0.0148 (17)	0.018 (2)	0.0005 (14)	0.0012 (15)	−0.0015 (15)
C5	0.0159 (18)	0.0174 (18)	0.0179 (19)	0.0000 (14)	0.0011 (14)	−0.0027 (14)
C6	0.0158 (18)	0.0129 (16)	0.0160 (18)	0.0002 (13)	0.0005 (14)	0.0012 (13)
C7	0.0126 (17)	0.0239 (19)	0.0219 (19)	−0.0018 (14)	0.0060 (14)	0.0017 (15)
O6	0.0258 (14)	0.0178 (13)	0.0147 (13)	0.0015 (11)	0.0082 (11)	0.0001 (10)
O7	0.0216 (14)	0.0236 (13)	0.0220 (14)	0.0079 (11)	0.0061 (11)	0.0087 (11)
O8	0.0186 (13)	0.0195 (13)	0.0178 (14)	0.0053 (11)	0.0085 (11)	0.0013 (11)
O9	0.0195 (14)	0.0183 (13)	0.0198 (15)	0.0033 (11)	0.0022 (11)	0.0034 (11)
O10	0.0277 (15)	0.0321 (15)	0.0172 (14)	0.0166 (13)	0.0112 (12)	0.0091 (12)
N2	0.0138 (17)	0.0142 (15)	0.0156 (18)	0.0011 (12)	0.0033 (14)	−0.0003 (13)
C8	0.0174 (18)	0.0218 (19)	0.0137 (18)	−0.0021 (15)	0.0031 (15)	0.0016 (14)
C9	0.0158 (19)	0.0171 (18)	0.0101 (18)	−0.0024 (15)	−0.0007 (15)	0.0017 (14)
C10	0.0126 (19)	0.0195 (19)	0.015 (2)	0.0044 (15)	0.0004 (16)	0.0004 (15)
C11	0.0175 (19)	0.0197 (18)	0.0119 (19)	−0.0022 (15)	0.0048 (15)	0.0000 (15)
C12	0.0136 (18)	0.0149 (18)	0.0086 (17)	0.0021 (14)	0.0011 (14)	−0.0003 (13)
C13	0.0138 (19)	0.0131 (18)	0.015 (2)	−0.0035 (14)	0.0010 (16)	0.0011 (14)
C14	0.0121 (17)	0.0106 (17)	0.020 (2)	−0.0004 (14)	0.0035 (15)	−0.0019 (15)
Zn2	0.0154 (2)	0.0173 (2)	0.0139 (2)	0.00010 (18)	0.00470 (18)	0.00074 (18)
O11	0.0136 (12)	0.0253 (13)	0.0206 (13)	0.0047 (10)	0.0050 (10)	0.0058 (11)
O12	0.0290 (14)	0.0288 (14)	0.0389 (16)	0.0161 (12)	0.0088 (12)	0.0054 (12)
O13	0.0168 (15)	0.0226 (14)	0.0173 (15)	0.0064 (11)	0.0077 (12)	0.0060 (11)
O14	0.0158 (13)	0.0230 (14)	0.0245 (15)	0.0031 (11)	0.0077 (11)	0.0014 (11)
O15	0.0270 (15)	0.0259 (14)	0.0190 (14)	0.0017 (12)	0.0059 (11)	0.0054 (11)
N3	0.0107 (15)	0.0167 (15)	0.0164 (16)	0.0009 (12)	0.0022 (13)	0.0013 (12)
C15	0.0166 (17)	0.0193 (18)	0.027 (2)	0.0030 (14)	0.0029 (15)	−0.0009 (15)
C16	0.0121 (17)	0.0185 (18)	0.0161 (18)	−0.0037 (13)	0.0031 (14)	0.0002 (14)
C17	0.0174 (19)	0.0184 (18)	0.0175 (19)	−0.0024 (14)	−0.0014 (15)	−0.0013 (14)
C18	0.0199 (19)	0.0203 (18)	0.0108 (18)	−0.0099 (15)	0.0046 (14)	0.0016 (15)
C19	0.0138 (18)	0.0176 (18)	0.019 (2)	−0.0024 (14)	0.0049 (15)	−0.0027 (15)
C20	0.0110 (17)	0.0156 (17)	0.0136 (19)	−0.0029 (14)	0.0030 (14)	−0.0036 (14)
C21	0.0142 (19)	0.0151 (17)	0.0166 (19)	−0.0002 (15)	0.0005 (15)	−0.0001 (16)
O16	0.0205 (13)	0.0200 (13)	0.0151 (13)	−0.0034 (10)	0.0066 (10)	−0.0035 (10)
O17	0.0182 (13)	0.0217 (13)	0.0143 (12)	−0.0060 (10)	0.0017 (10)	−0.0027 (10)
O18	0.0174 (13)	0.0212 (14)	0.0175 (14)	−0.0040 (11)	0.0069 (11)	−0.0002 (11)
O19	0.0216 (14)	0.0296 (15)	0.0204 (15)	−0.0100 (12)	0.0054 (12)	−0.0081 (12)
O20	0.0227 (14)	0.0250 (13)	0.0175 (14)	−0.0117 (11)	0.0100 (11)	−0.0058 (11)
N4	0.0140 (16)	0.0165 (16)	0.0088 (16)	0.0002 (13)	0.0015 (13)	−0.0002 (12)
C22	0.0164 (18)	0.0157 (17)	0.0163 (19)	0.0008 (14)	−0.0010 (15)	0.0005 (14)
C23	0.0122 (18)	0.0100 (16)	0.0163 (19)	−0.0009 (14)	0.0000 (15)	0.0025 (14)
C24	0.0145 (19)	0.0126 (17)	0.016 (2)	−0.0009 (15)	0.0025 (16)	−0.0018 (14)
C25	0.0131 (19)	0.0181 (18)	0.0148 (19)	−0.0013 (14)	0.0026 (15)	0.0022 (15)
C26	0.0147 (19)	0.0146 (18)	0.016 (2)	0.0007 (14)	0.0013 (15)	−0.0042 (14)
C27	0.0102 (18)	0.0130 (18)	0.013 (2)	−0.0021 (14)	−0.0014 (15)	0.0024 (14)
C28	0.0132 (18)	0.0172 (18)	0.0170 (19)	0.0023 (15)	0.0027 (15)	−0.0020 (15)
N5	0.0171 (15)	0.0196 (15)	0.0254 (18)	0.0002 (12)	0.0016 (13)	−0.0045 (13)
N6	0.0213 (15)	0.0205 (14)	0.0260 (15)	0.0038 (12)	0.0069 (12)	0.0013 (12)
C29	0.0233 (18)	0.0172 (17)	0.0216 (17)	−0.0001 (14)	0.0030 (14)	0.0024 (14)
C30	0.0160 (17)	0.0241 (18)	0.026 (2)	0.0000 (14)	0.0006 (15)	0.0039 (15)
C31	0.0167 (18)	0.0242 (19)	0.0222 (19)	0.0006 (15)	0.0002 (15)	0.0004 (15)
N7	0.0214 (16)	0.0316 (17)	0.0272 (17)	0.0113 (14)	0.0080 (13)	0.0018 (14)
N8	0.0185 (16)	0.0197 (15)	0.0299 (19)	0.0001 (12)	−0.0005 (13)	−0.0035 (13)
C32	0.0252 (19)	0.031 (2)	0.0220 (18)	0.0004 (16)	0.0073 (15)	−0.0004 (15)
C33	0.0190 (18)	0.0220 (17)	0.0216 (18)	0.0035 (15)	0.0005 (14)	−0.0013 (15)
C34	0.0241 (18)	0.0264 (18)	0.0170 (17)	−0.0012 (15)	0.0017 (14)	−0.0004 (14)
O1W	0.0332 (15)	0.0187 (14)	0.0200 (15)	−0.0064 (12)	0.0090 (12)	−0.0002 (11)
O2W	0.0331 (16)	0.0172 (13)	0.0185 (15)	0.0068 (11)	0.0044 (12)	0.0023 (11)
O3W	0.0301 (14)	0.0278 (14)	0.0491 (17)	−0.0086 (12)	0.0184 (13)	−0.0109 (12)
O4W	0.0255 (14)	0.0562 (18)	0.0217 (13)	0.0094 (13)	0.0067 (11)	−0.0025 (13)
O5W	0.0251 (13)	0.0329 (14)	0.0172 (12)	−0.0035 (11)	−0.0009 (10)	0.0069 (10)
O6W	0.0275 (14)	0.0194 (13)	0.0301 (15)	0.0026 (11)	0.0126 (12)	0.0022 (11)
O7W	0.0204 (13)	0.0264 (13)	0.0277 (15)	−0.0008 (11)	0.0058 (11)	−0.0004 (11)

### Geometric parameters (Å, °)

**Table:**

Zn1—N1	2.012 (3)	O17—C22	1.259 (4)
Zn1—N2	1.999 (3)	O18—C28	1.271 (4)
Zn1—O1	2.081 (3)	O19—C28	1.246 (4)
Zn1—O3	2.424 (3)	O20—C25	1.340 (5)
Zn1—O6	2.155 (3)	O20—H20O	0.8504
Zn1—O8	2.201 (3)	N4—C23	1.331 (5)
O1—C1	1.282 (5)	N4—C27	1.340 (5)
O2—C1	1.247 (5)	C22—C23	1.533 (5)
O3—C7	1.257 (4)	C23—C24	1.362 (5)
O4—C7	1.254 (4)	C24—C25	1.401 (5)
O5—C4	1.330 (5)	C24—H24A	0.9300
O5—H5O	0.82 (3)	C25—C26	1.390 (5)
N1—C6	1.345 (4)	C26—C27	1.378 (6)
N1—C2	1.345 (5)	C26—H26A	0.9300
C1—C2	1.508 (5)	C27—C28	1.506 (5)
C2—C3	1.374 (5)	N5—C29	1.497 (4)
C3—C4	1.399 (5)	N5—H5NA	0.8599
C3—H3A	0.9300	N5—H5NB	0.8600
C4—C5	1.404 (5)	N5—H5NC	0.8601
C5—C6	1.377 (5)	N6—C31	1.489 (4)
C5—H5A	0.9300	N6—H6NA	0.8601
C6—C7	1.519 (5)	N6—H6NB	0.8600
O6—C8	1.259 (4)	N6—H6NC	0.8599
O7—C8	1.256 (5)	C29—C30	1.501 (4)
O8—C14	1.283 (4)	C29—H29A	0.9700
O9—C14	1.235 (4)	C29—H29B	0.9700
O10—C11	1.349 (5)	C30—C31	1.528 (5)
O10—H10O	0.8881	C30—H30A	0.9700
N2—C9	1.335 (5)	C30—H30B	0.9700
N2—C13	1.345 (5)	C31—H31A	0.9700
C8—C9	1.510 (5)	C31—H31B	0.9700
C9—C10	1.394 (5)	N7—C32	1.488 (4)
C10—C11	1.412 (5)	N7—H7NA	0.8601
C10—H10A	0.9300	N7—H7NB	0.8600
C11—C12	1.386 (5)	N7—H7NC	0.8599
C12—C13	1.380 (5)	N8—C34	1.475 (4)
C12—H12A	0.9300	N8—H8NA	0.8601
C13—C14	1.518 (5)	N8—H8NB	0.8600
Zn2—N3	2.001 (3)	N8—H8NC	0.8598
Zn2—N4	2.007 (3)	C32—C33	1.512 (5)
Zn2—O11	2.331 (2)	C32—H32A	0.9700
Zn2—O13	2.135 (3)	C32—H32B	0.9700
Zn2—O16	2.234 (3)	C33—C34	1.510 (5)
Zn2—O18	2.149 (3)	C33—H33A	0.9700
O11—C15	1.283 (4)	C33—H33B	0.9700
O12—C15	1.229 (4)	C34—H34A	0.9700
O13—C21	1.272 (5)	C34—H34B	0.9700
O14—C21	1.244 (5)	O1W—H1WA	0.81 (3)
O15—C18	1.351 (4)	O1W—H1WB	0.86 (3)
O15—H15O	0.88 (2)	O2W—H2WA	0.84 (3)
N3—C16	1.340 (4)	O2W—H2WB	0.89 (3)
N3—C20	1.344 (5)	O3W—H3WA	0.8200
C15—C16	1.526 (5)	O3W—H3WB	0.8199
C16—C17	1.376 (5)	O4W—H4WA	0.8200
C17—C18	1.389 (5)	O4W—H4WB	0.8200
C17—H17A	0.9300	O5W—H5WA	0.8199
C18—C19	1.391 (5)	O5W—H5WB	0.8200
C19—C20	1.384 (5)	O6W—H6WA	0.8200
C19—H19A	0.9300	O6W—H6WB	0.8200
C20—C21	1.513 (5)	O7W—H7WA	0.8201
O16—C22	1.260 (4)	O7W—H7WB	0.8200
			
N2—Zn1—N1	157.03 (13)	C20—C19—H19A	120.6
N2—Zn1—O1	124.02 (12)	C18—C19—H19A	120.6
N1—Zn1—O1	78.86 (12)	N3—C20—C19	121.6 (3)
N2—Zn1—O6	76.72 (11)	N3—C20—C21	113.5 (3)
N1—Zn1—O6	105.64 (11)	C19—C20—C21	124.8 (3)
O1—Zn1—O6	94.67 (10)	O14—C21—O13	125.3 (4)
N2—Zn1—O8	76.36 (11)	O14—C21—C20	119.4 (3)
N1—Zn1—O8	102.43 (11)	O13—C21—C20	115.2 (3)
O1—Zn1—O8	93.39 (10)	C22—O16—Zn2	113.5 (2)
O6—Zn1—O8	151.79 (10)	C28—O18—Zn2	115.0 (2)
N2—Zn1—O3	84.80 (11)	C25—O20—H20O	118.6
N1—Zn1—O3	72.27 (11)	C23—N4—C27	120.3 (3)
O1—Zn1—O3	150.12 (9)	C23—N4—Zn2	120.0 (3)
O6—Zn1—O3	100.28 (9)	C27—N4—Zn2	117.9 (2)
O8—Zn1—O3	85.54 (9)	O17—C22—O16	125.6 (3)
C1—O1—Zn1	115.0 (2)	O17—C22—C23	118.3 (3)
C7—O3—Zn1	111.5 (2)	O16—C22—C23	116.1 (3)
C4—O5—H5O	118 (3)	N4—C23—C24	121.9 (3)
C6—N1—C2	119.6 (3)	N4—C23—C22	112.6 (3)
C6—N1—Zn1	124.1 (2)	C24—C23—C22	125.5 (3)
C2—N1—Zn1	116.2 (2)	C23—C24—C25	118.5 (3)
O2—C1—O1	124.6 (4)	C23—C24—H24A	120.7
O2—C1—C2	119.5 (4)	C25—C24—H24A	120.7
O1—C1—C2	115.8 (3)	O20—C25—C26	118.4 (4)
N1—C2—C3	121.9 (3)	O20—C25—C24	122.1 (3)
N1—C2—C1	112.8 (3)	C26—C25—C24	119.6 (4)
C3—C2—C1	125.2 (3)	C27—C26—C25	117.9 (4)
C2—C3—C4	119.2 (4)	C27—C26—H26A	121.0
C2—C3—H3A	120.4	C25—C26—H26A	121.0
C4—C3—H3A	120.4	N4—C27—C26	121.8 (3)
O5—C4—C3	124.0 (3)	N4—C27—C28	113.1 (3)
O5—C4—C5	117.7 (3)	C26—C27—C28	125.1 (3)
C3—C4—C5	118.2 (3)	O19—C28—O18	124.8 (4)
C6—C5—C4	119.0 (4)	O19—C28—C27	119.6 (3)
C6—C5—H5A	120.5	O18—C28—C27	115.5 (3)
C4—C5—H5A	120.5	C29—N5—H5NA	115.7
N1—C6—C5	121.7 (3)	C29—N5—H5NB	105.8
N1—C6—C7	113.7 (3)	H5NA—N5—H5NB	87.5
C5—C6—C7	124.5 (3)	C29—N5—H5NC	114.7
O4—C7—O3	126.5 (3)	H5NA—N5—H5NC	114.9
O4—C7—C6	117.4 (3)	H5NB—N5—H5NC	114.9
O3—C7—C6	115.8 (3)	C31—N6—H6NA	106.5
C8—O6—Zn1	114.8 (2)	C31—N6—H6NB	109.8
C14—O8—Zn1	114.3 (2)	H6NA—N6—H6NB	114.9
C11—O10—H10O	111.1	C31—N6—H6NC	100.1
C9—N2—C13	120.7 (3)	H6NA—N6—H6NC	114.0
C9—N2—Zn1	118.8 (3)	H6NB—N6—H6NC	110.4
C13—N2—Zn1	119.7 (3)	N5—C29—C30	110.7 (3)
O7—C8—O6	125.9 (4)	N5—C29—H29A	109.5
O7—C8—C9	118.2 (3)	C30—C29—H29A	109.5
O6—C8—C9	115.9 (3)	N5—C29—H29B	109.5
N2—C9—C10	121.5 (3)	C30—C29—H29B	109.5
N2—C9—C8	112.9 (3)	H29A—C29—H29B	108.1
C10—C9—C8	125.6 (3)	C29—C30—C31	114.7 (3)
C9—C10—C11	117.3 (3)	C29—C30—H30A	108.6
C9—C10—H10A	121.3	C31—C30—H30A	108.6
C11—C10—H10A	121.3	C29—C30—H30B	108.6
O10—C11—C12	118.6 (4)	C31—C30—H30B	108.6
O10—C11—C10	120.9 (3)	H30A—C30—H30B	107.6
C12—C11—C10	120.5 (4)	N6—C31—C30	113.8 (3)
C13—C12—C11	118.0 (4)	N6—C31—H31A	108.8
C13—C12—H12A	121.0	C30—C31—H31A	108.8
C11—C12—H12A	121.0	N6—C31—H31B	108.8
N2—C13—C12	121.9 (3)	C30—C31—H31B	108.8
N2—C13—C14	113.1 (3)	H31A—C31—H31B	107.7
C12—C13—C14	125.0 (3)	C32—N7—H7NA	96.1
O9—C14—O8	125.3 (3)	C32—N7—H7NB	100.6
O9—C14—C13	119.2 (3)	H7NA—N7—H7NB	118.2
O8—C14—C13	115.5 (3)	C32—N7—H7NC	104.9
N3—Zn2—N4	160.29 (12)	H7NA—N7—H7NC	139.3
N3—Zn2—O13	77.85 (11)	H7NB—N7—H7NC	91.9
N4—Zn2—O13	121.86 (11)	C34—N8—H8NA	100.1
N3—Zn2—O18	103.62 (11)	C34—N8—H8NB	110.0
N4—Zn2—O18	76.93 (11)	H8NA—N8—H8NB	111.4
O13—Zn2—O18	95.44 (10)	C34—N8—H8NC	104.7
N3—Zn2—O16	105.86 (11)	H8NA—N8—H8NC	117.7
N4—Zn2—O16	75.45 (11)	H8NB—N8—H8NC	111.9
O13—Zn2—O16	90.48 (10)	N7—C32—C33	111.3 (3)
O18—Zn2—O16	150.52 (10)	N7—C32—H32A	109.4
N3—Zn2—O11	74.19 (10)	C33—C32—H32A	109.4
N4—Zn2—O11	86.11 (11)	N7—C32—H32B	109.4
O13—Zn2—O11	152.03 (10)	C33—C32—H32B	109.4
O18—Zn2—O11	90.70 (10)	H32A—C32—H32B	108.0
O16—Zn2—O11	97.49 (9)	C34—C33—C32	110.8 (3)
C15—O11—Zn2	113.1 (2)	C34—C33—H33A	109.5
C21—O13—Zn2	115.0 (2)	C32—C33—H33A	109.5
C18—O15—H15O	111 (2)	C34—C33—H33B	109.5
C16—N3—C20	119.4 (3)	C32—C33—H33B	109.5
C16—N3—Zn2	123.0 (2)	H33A—C33—H33B	108.1
C20—N3—Zn2	117.6 (2)	N8—C34—C33	112.4 (3)
O12—C15—O11	127.3 (3)	N8—C34—H34A	109.1
O12—C15—C16	117.4 (3)	C33—C34—H34A	109.1
O11—C15—C16	115.2 (3)	N8—C34—H34B	109.1
N3—C16—C17	122.4 (3)	C33—C34—H34B	109.1
N3—C16—C15	114.2 (3)	H34A—C34—H34B	107.9
C17—C16—C15	123.3 (3)	H1WA—O1W—H1WB	102 (4)
C16—C17—C18	118.4 (4)	H2WA—O2W—H2WB	100 (4)
C16—C17—H17A	120.8	H3WA—O3W—H3WB	93.0
C18—C17—H17A	120.8	H4WA—O4W—H4WB	106.5
O15—C18—C17	117.4 (3)	H5WA—O5W—H5WB	112.4
O15—C18—C19	123.2 (3)	H6WA—O6W—H6WB	126.0
C17—C18—C19	119.4 (3)	H7WA—O7W—H7WB	97.0
C20—C19—C18	118.7 (3)		
			
N2—Zn1—O1—C1	179.8 (2)	O13—Zn2—O11—C15	3.0 (4)
N1—Zn1—O1—C1	2.0 (3)	O18—Zn2—O11—C15	−100.0 (2)
O6—Zn1—O1—C1	−103.1 (3)	O16—Zn2—O11—C15	108.4 (2)
O8—Zn1—O1—C1	104.0 (3)	N3—Zn2—O13—C21	−3.4 (3)
O3—Zn1—O1—C1	17.0 (4)	N4—Zn2—O13—C21	177.4 (3)
N2—Zn1—O3—C7	−174.3 (2)	O18—Zn2—O13—C21	99.4 (3)
N1—Zn1—O3—C7	6.9 (2)	O16—Zn2—O13—C21	−109.5 (3)
O1—Zn1—O3—C7	−8.6 (3)	O11—Zn2—O13—C21	−2.4 (4)
O6—Zn1—O3—C7	110.2 (2)	N4—Zn2—N3—C16	−2.9 (5)
O8—Zn1—O3—C7	−97.7 (2)	O13—Zn2—N3—C16	179.0 (3)
N2—Zn1—N1—C6	1.0 (5)	O18—Zn2—N3—C16	86.3 (3)
O1—Zn1—N1—C6	176.4 (3)	O16—Zn2—N3—C16	−94.0 (3)
O6—Zn1—N1—C6	−91.9 (3)	O11—Zn2—N3—C16	−0.5 (3)
O8—Zn1—N1—C6	85.3 (3)	N4—Zn2—N3—C20	175.3 (3)
O3—Zn1—N1—C6	4.2 (3)	O13—Zn2—N3—C20	−2.8 (2)
N2—Zn1—N1—C2	176.2 (3)	O18—Zn2—N3—C20	−95.5 (3)
O1—Zn1—N1—C2	−8.4 (2)	O16—Zn2—N3—C20	84.2 (3)
O6—Zn1—N1—C2	83.3 (3)	O11—Zn2—N3—C20	177.7 (3)
O8—Zn1—N1—C2	−99.5 (3)	Zn2—O11—C15—O12	172.8 (3)
O3—Zn1—N1—C2	179.4 (3)	Zn2—O11—C15—C16	−6.2 (4)
Zn1—O1—C1—O2	−171.5 (3)	C20—N3—C16—C17	−2.2 (5)
Zn1—O1—C1—C2	4.1 (4)	Zn2—N3—C16—C17	175.9 (3)
C6—N1—C2—C3	4.8 (5)	C20—N3—C16—C15	179.4 (3)
Zn1—N1—C2—C3	−170.6 (3)	Zn2—N3—C16—C15	−2.5 (4)
C6—N1—C2—C1	−171.9 (3)	O12—C15—C16—N3	−173.1 (3)
Zn1—N1—C2—C1	12.7 (4)	O11—C15—C16—N3	6.0 (4)
O2—C1—C2—N1	164.8 (3)	O12—C15—C16—C17	8.5 (5)
O1—C1—C2—N1	−11.0 (5)	O11—C15—C16—C17	−172.4 (3)
O2—C1—C2—C3	−11.8 (6)	N3—C16—C17—C18	−0.4 (5)
O1—C1—C2—C3	172.5 (3)	C15—C16—C17—C18	177.8 (3)
N1—C2—C3—C4	−0.2 (5)	C16—C17—C18—O15	−178.3 (3)
C1—C2—C3—C4	176.0 (3)	C16—C17—C18—C19	1.6 (5)
C2—C3—C4—O5	178.2 (4)	O15—C18—C19—C20	179.7 (3)
C2—C3—C4—C5	−4.5 (5)	C17—C18—C19—C20	−0.2 (5)
O5—C4—C5—C6	−177.8 (3)	C16—N3—C20—C19	3.7 (5)
C3—C4—C5—C6	4.8 (5)	Zn2—N3—C20—C19	−174.5 (3)
C2—N1—C6—C5	−4.5 (5)	C16—N3—C20—C21	−174.2 (3)
Zn1—N1—C6—C5	170.6 (3)	Zn2—N3—C20—C21	7.6 (4)
C2—N1—C6—C7	171.9 (3)	C18—C19—C20—N3	−2.5 (5)
Zn1—N1—C6—C7	−13.1 (4)	C18—C19—C20—C21	175.1 (3)
C4—C5—C6—N1	−0.3 (5)	Zn2—O13—C21—O14	−168.6 (3)
C4—C5—C6—C7	−176.3 (3)	Zn2—O13—C21—C20	8.2 (4)
Zn1—O3—C7—O4	159.6 (3)	N3—C20—C21—O14	166.5 (3)
Zn1—O3—C7—C6	−15.2 (4)	C19—C20—C21—O14	−11.3 (6)
N1—C6—C7—O4	−156.3 (3)	N3—C20—C21—O13	−10.5 (5)
C5—C6—C7—O4	19.9 (5)	C19—C20—C21—O13	171.7 (3)
N1—C6—C7—O3	18.9 (4)	N3—Zn2—O16—C22	148.1 (2)
C5—C6—C7—O3	−164.8 (3)	N4—Zn2—O16—C22	−11.5 (2)
N2—Zn1—O6—C8	−7.6 (3)	O13—Zn2—O16—C22	−134.4 (2)
N1—Zn1—O6—C8	148.8 (3)	O18—Zn2—O16—C22	−32.4 (3)
O1—Zn1—O6—C8	−131.5 (3)	O11—Zn2—O16—C22	72.5 (2)
O8—Zn1—O6—C8	−25.3 (4)	N3—Zn2—O18—C28	−154.0 (3)
O3—Zn1—O6—C8	74.5 (3)	N4—Zn2—O18—C28	5.7 (3)
N2—Zn1—O8—C14	5.6 (2)	O13—Zn2—O18—C28	127.2 (3)
N1—Zn1—O8—C14	−150.9 (2)	O16—Zn2—O18—C28	26.5 (4)
O1—Zn1—O8—C14	129.8 (2)	O11—Zn2—O18—C28	−80.1 (3)
O6—Zn1—O8—C14	23.4 (4)	N3—Zn2—N4—C23	−81.9 (5)
O3—Zn1—O8—C14	−80.1 (2)	O13—Zn2—N4—C23	95.9 (3)
N1—Zn1—N2—C9	−90.0 (4)	O18—Zn2—N4—C23	−175.8 (3)
O1—Zn1—N2—C9	95.5 (3)	O16—Zn2—N4—C23	14.6 (3)
O6—Zn1—N2—C9	8.8 (3)	O11—Zn2—N4—C23	−84.2 (3)
O8—Zn1—N2—C9	−179.7 (3)	N3—Zn2—N4—C27	82.8 (5)
O3—Zn1—N2—C9	−93.1 (3)	O13—Zn2—N4—C27	−99.5 (3)
N1—Zn1—N2—C13	80.0 (4)	O18—Zn2—N4—C27	−11.2 (3)
O1—Zn1—N2—C13	−94.5 (3)	O16—Zn2—N4—C27	179.2 (3)
O6—Zn1—N2—C13	178.8 (3)	O11—Zn2—N4—C27	80.4 (3)
O8—Zn1—N2—C13	−9.7 (3)	Zn2—O16—C22—O17	−174.4 (3)
O3—Zn1—N2—C13	77.0 (3)	Zn2—O16—C22—C23	7.3 (4)
Zn1—O6—C8—O7	−176.1 (3)	C27—N4—C23—C24	−0.9 (5)
Zn1—O6—C8—C9	5.3 (4)	Zn2—N4—C23—C24	163.4 (3)
C13—N2—C9—C10	1.2 (5)	C27—N4—C23—C22	−179.5 (3)
Zn1—N2—C9—C10	171.1 (3)	Zn2—N4—C23—C22	−15.2 (4)
C13—N2—C9—C8	−178.6 (3)	O17—C22—C23—N4	−174.4 (3)
Zn1—N2—C9—C8	−8.7 (4)	O16—C22—C23—N4	4.0 (4)
O7—C8—C9—N2	−176.9 (3)	O17—C22—C23—C24	7.0 (5)
O6—C8—C9—N2	1.7 (5)	O16—C22—C23—C24	−174.5 (3)
O7—C8—C9—C10	3.2 (6)	N4—C23—C24—C25	0.2 (5)
O6—C8—C9—C10	−178.1 (4)	C22—C23—C24—C25	178.5 (3)
N2—C9—C10—C11	−3.0 (5)	C23—C24—C25—O20	179.6 (3)
C8—C9—C10—C11	176.9 (3)	C23—C24—C25—C26	0.8 (5)
C9—C10—C11—O10	−179.6 (3)	O20—C25—C26—C27	−179.8 (3)
C9—C10—C11—C12	2.5 (5)	C24—C25—C26—C27	−1.1 (5)
O10—C11—C12—C13	−178.3 (3)	C23—N4—C27—C26	0.7 (5)
C10—C11—C12—C13	−0.4 (5)	Zn2—N4—C27—C26	−163.9 (3)
C9—N2—C13—C12	1.1 (5)	C23—N4—C27—C28	179.0 (3)
Zn1—N2—C13—C12	−168.7 (3)	Zn2—N4—C27—C28	14.4 (4)
C9—N2—C13—C14	−178.3 (3)	C25—C26—C27—N4	0.3 (5)
Zn1—N2—C13—C14	11.9 (4)	C25—C26—C27—C28	−177.8 (3)
C11—C12—C13—N2	−1.5 (5)	Zn2—O18—C28—O19	−177.3 (3)
C11—C12—C13—C14	177.8 (3)	Zn2—O18—C28—C27	0.0 (4)
Zn1—O8—C14—O9	−179.1 (3)	N4—C27—C28—O19	168.4 (3)
Zn1—O8—C14—C13	−1.3 (4)	C26—C27—C28—O19	−13.3 (6)
N2—C13—C14—O9	171.6 (3)	N4—C27—C28—O18	−9.1 (5)
C12—C13—C14—O9	−7.8 (5)	C26—C27—C28—O18	169.2 (3)
N2—C13—C14—O8	−6.4 (4)	N5—C29—C30—C31	−175.7 (3)
C12—C13—C14—O8	174.3 (3)	C29—C30—C31—N6	−81.2 (4)
N3—Zn2—O11—C15	3.9 (2)	N7—C32—C33—C34	176.7 (3)
N4—Zn2—O11—C15	−176.9 (2)	C32—C33—C34—N8	169.1 (3)

### Hydrogen-bond geometry (Å, °)

**Table:**

*D*—H···*A*	*D*—H	H···*A*	*D*···*A*	*D*—H···*A*
O5—H5O···O1W^i^	0.82 (4)	1.79 (4)	2.577 (5)	162 (3)
O10—H10O···O12^ii^	0.89	1.73	2.613 (4)	170
O15—H15O···O2W^iii^	0.88 (4)	1.73 (4)	2.600 (4)	170 (4)
O20—H20O···O4	0.85	1.80	2.600 (3)	156
N5—H5NA···O14^iv^	0.86	2.16	3.019 (4)	176
N5—H5NB···O4W	0.86	1.94	2.804 (4)	176
N5—H5NC···O4^v^	0.86	2.23	3.019 (4)	152
N5—H5NC···O7W^v^	0.86	2.44	2.843 (4)	110
N6—H6NA···O14^vi^	0.86	2.03	2.870 (4)	164
N6—H6NB···O5W^i^	0.86	1.94	2.796 (3)	174
N6—H6NC···O9^vii^	0.86	1.93	2.771 (4)	164
N7—H7NA···O16^vi^	0.86	2.15	2.943 (4)	153
N7—H7NB···O19^iv^	0.86	1.99	2.714 (4)	141
N7—H7NC···O9^vii^	0.86	2.14	2.991 (4)	169
N8—H8NA···O6W^vi^	0.86	1.89	2.724 (4)	164
N8—H8NB···O2^vii^	0.86	2.00	2.828 (4)	160
N8—H8NC···O3W^viii^	0.86	1.94	2.791 (4)	169
O1W—H1WA···O18^ix^	0.82 (4)	1.90 (4)	2.701 (4)	164 (4)
O1W—H1WB···O1	0.86 (3)	1.84 (4)	2.690 (4)	171 (4)
O2W—H2WA···O8	0.83 (4)	1.94 (4)	2.738 (4)	163 (3)
O2W—H2WB···O13^x^	0.89 (3)	1.81 (3)	2.688 (4)	171 (3)
O3W—H3WA···O17^i^	0.82	2.05	2.873 (4)	179
O3W—H3WB···O3^i^	0.82	2.14	2.950 (3)	172
O4W—H4WA···O20	0.82	2.32	2.876 (4)	126
O4W—H4WB···O11^v^	0.82	2.06	2.862 (3)	167
O5W—H5WA···O7	0.82	1.94	2.717 (3)	158
O5W—H5WB···O17^i^	0.82	2.05	2.846 (3)	165
O6W—H6WA···O11^ii^	0.82	1.94	2.755 (4)	169
O6W—H6WB···O7	0.82	1.96	2.726 (3)	154
O7W—H7WA···O3	0.82	2.03	2.829 (3)	164
O7W—H7WB···O17	0.82	2.14	2.953 (3)	170
C12—H12A···O6^xi^	0.93	2.33	3.209 (5)	157
C26—H26A···O16^v^	0.93	2.56	3.448 (5)	160
C31—H31A···O6^i^	0.97	2.54	3.398 (5)	148
C31—H31B···O19^iv^	0.97	2.44	3.400 (4)	170
C32—H32B···O2^i^	0.97	2.37	3.213 (4)	145

Symmetry codes: (i) *x*, −*y*+1, *z*−1/2; (ii) *x*, *y*+1, *z*; (iii) *x*−1, −*y*, *z*+1/2;  (iv) *x*+1, −*y*, *z*−1/2; (v) *x*, −*y*, *z*−1/2; (vi) *x*+1, *y*, *z*−1; (vii) *x*, *y*, *z*−1; (viii) *x*+1, −*y*+1, *z*−1/2; (ix) *x*+1, *y*+1, *z*; (x) *x*+1, *y*, *z*; (xi) *x*, −*y*+1, *z*+1/2.
